# Bleeding events associated with fibrinolytic therapy and primary percutaneous coronary intervention in patients with STEMI: A systematic review and meta-analysis of randomized controlled trials

**DOI:** 10.1097/MD.0000000000003877

**Published:** 2016-06-10

**Authors:** Pravesh Kumar Bundhun, Girish Janoo, Meng-Hua Chen

**Affiliations:** Institute of Cardiovascular Diseases, the First Affiliated Hospital of Guangxi Medical University, Nanning, Guangxi, P. R. China.

**Keywords:** bleeding complications, fibrinolysis, primary angioplasty, ST segment elevated myocardial infarction

## Abstract

From the year 1986 onwards, several studies have been published focusing on the comparison between fibrinolysis and primary percutaneous coronary intervention (PPCI) in patients with ST segment elevated myocardial infarction (STEMI). However, because antiplatelet and anticoagulating medications are used in approximation, before and during these procedures, bleeding events have been reported to be associated with both reperfusion therapies. This study aimed to compare the bleeding events associated with fibrinolytic therapy and primary angioplasty in patients with STEMI. Randomized controlled trials (RCTs) comparing fibrinolysis and primary angioplasty in patients with STEMI were searched from Medline, PubMed, EMBASE, and the Cochrane databases. Bleeding complications following 30 days from hospitalization were considered as the primary clinical endpoints in this study. Secondary endpoints included all-cause mortality, re-infarction, stroke, and shock. Antiplatelet and anticoagulating drugs used during these 2 different procedures were compared. Odds ratios (ORs) with 95% confidence intervals (CIs) were calculated and the pooled analyses were performed with RevMan 5.3 software. Twelve studies involving 10 RCTs consisting of a total number of 5561 patients (2784 patients from the fibrinolysis group and 2777 patients from the PPCI group) were included in this meta-analysis. Our results showed no significant difference in the overall bleeding complications during a 30-day period between these 2 reperfusion therapies with OR 1.02; 95% CI 0.89 to 1.17, *P* = 0.78. Nonintracranial bleeding was also not statistically significant with OR 0.85; 95% CI 0.70 to 1.04, *P* = 0.12. However, fibrinolytic therapy was associated with a significantly higher rate of intracranial bleeding with OR 0.17; 95% CI 0.06 to 0.50, *P* = 0.001 than PPCI. In addition, death, re-infarction, and stroke significantly favored primary angioplasty. According to the results of this study, even if the rate of nonintracranial bleeding was not statistically significant between these 2 reperfusion therapies, fibrinolytic therapy was associated with a significantly higher rate of intracranial bleeding than PPCI. In addition, PPCI was associated with a significantly lower rate of death, reinfarction, and stroke. Therefore, PPCI should be recommended in patients with STEMI, especially in PCI-capable hospitals.

## 1 Introduction

Guidelines normally recommend primary percutaneous coronary intervention (PPCI) as the main reperfusion therapy in patients with ST segment elevated myocardial infarction (STEMI) treated in PCI-capable hospitals, whereas if not contraindicated, fibrinolytic therapy has been the preferred reperfusion therapy for similar patients in non-PCI capable centers.^[1]^

Several studies have shown an increased risk of bleeding events associated with fibrinolytic therapy.^[2]^ Unfortunately, PPCI is also not completely safe. Even if the risk of myocardial infarction (MI) is reduced in patients treated by PCI, platelet inhibition increases the risk of bleeding in these patients after primary angioplasty.^[3]^

From the year 1986 onwards, several studies have been published focusing on the comparison between fibrinolysis and PPCI in patients with STEMI. However, because antiplatelet and anticoagulating medications are used in approximation, before and during these procedures, for example, medications are often given according to weight and age, which are not often accurate, bleeding has been reported with both reperfusion therapies. As very few studies have assessed bleeding rates manifested between these 2 reperfusion therapies, this study aimed to compare the bleeding events associated with fibrinolytic therapy and primary angioplasty in patients with STEMI.

## 2 Methods

### 2.1 Data sources and searched strategy

Medline, PubMed, EMBASE, and Cochrane databases were searched for RCTs comparing PPCI with fibrinolysis by typing the words or phrase “primary percutaneous coronary intervention and fibrinolysis and STEMI.” The abbreviation “PPCI” was also used. To further enhance this search, PPCI was also replaced by the word “primary angioplasty.” Relevant reference lists were also searched for suitable RCTs. Publications written in English were considered in this search strategy.

### 2.2 Inclusion and exclusion criteria

RCTs were included if:

1. They compared PPCI with fibrinolysis.
2. Bleeding complications were reported among their clinical outcomes.
3. They had a follow-up period of less than or equal to 30 days.

RCTs were excluded if:

1. They did not compare PPCI with fibrinolysis.
2. Outcomes related to bleeding were not reported.
3. They had a longer follow-up period.
4. They were duplicates.

### 2.3 Eligibility of the participants, the antiplatelet agents, and anticoagulating agents used in patients randomized to fibrinolysis and PPCI, respectively

Patients were eligible for enrollment if:

1. They arrived at the hospital within 3 hours after the onset of symptoms related to MI.
2. Results from their electrocardiogram showed evidence of acute STEMI represented by at least 2 mm elevation of the ST segment in 2 contiguous precordial leads.
3. PPCI could not be initiated within a period of about 1 hour after their first medical contact.

Patients were not eligible for enrollment if:

1. They had a history of hemorrhagic diathesis or any contraindication to fibrinolysis.
2. They had severe renal or hepatic insufficiency.
3. They had a history of previous aorto-femoral bypass or any condition that could hamper femoral artery access.
4. They had a history of cardiogenic shock or coronary artery bypass surgery (CABG).
5. They had previously been receiving oral anticoagulation therapy.

Antiplatelet and anticoagulating medications were used before and during these reperfusion procedures (PPCI and fibrinolysis, respectively). For example, an infusion of at least 5000 U heparin was required in the beginning in both groups.

Those patients randomized for primary angioplasty required at least another 5000 U intravenous infusion of heparin before catheterization. In the beginning, all patients were given at least 300 mg Aspirin. Clopidogrel 75 mg/day, ticlopidine 500 mg/day, and glycoprotein iib/iiia were also used before catheterization depending on the choice of the physician and the condition of the patients. These antiplatelets were continued orally for 1 month after reperfusion in certain trials.

For those patients who were randomized to fibrinolytic therapy, in addition to 5000 U heparin and 300 mg aspirin, tissue plasminogen activator such as alteplase, tenecteplase, activase, or streptokinase were also infused. Fifteen milligram alteplase followed by an alteplase infusion of 0.75 mg/kg (not exceeding 50 mg) over 30 minutes and then 0.5 mg/kg (not exceeding 35 mg) over the next 60 minutes up to a total dose of 100 mg were required in some trials. Other trials involved the use of 1_**�**_5 million U streptokinase intravenously over a period of 1 hour or activase given at a dose of 100 mg over 3 hours intravenously.

An additional heparin infusion was given for 3 to 5 days to patients from both groups. Medication dosage was given according to guidelines. Dosage could vary from 1 patient to another depending on their total weight and age. Detailed information about the blood thinners and anticoagulants have been provided in Table 1.

**Table 1:**
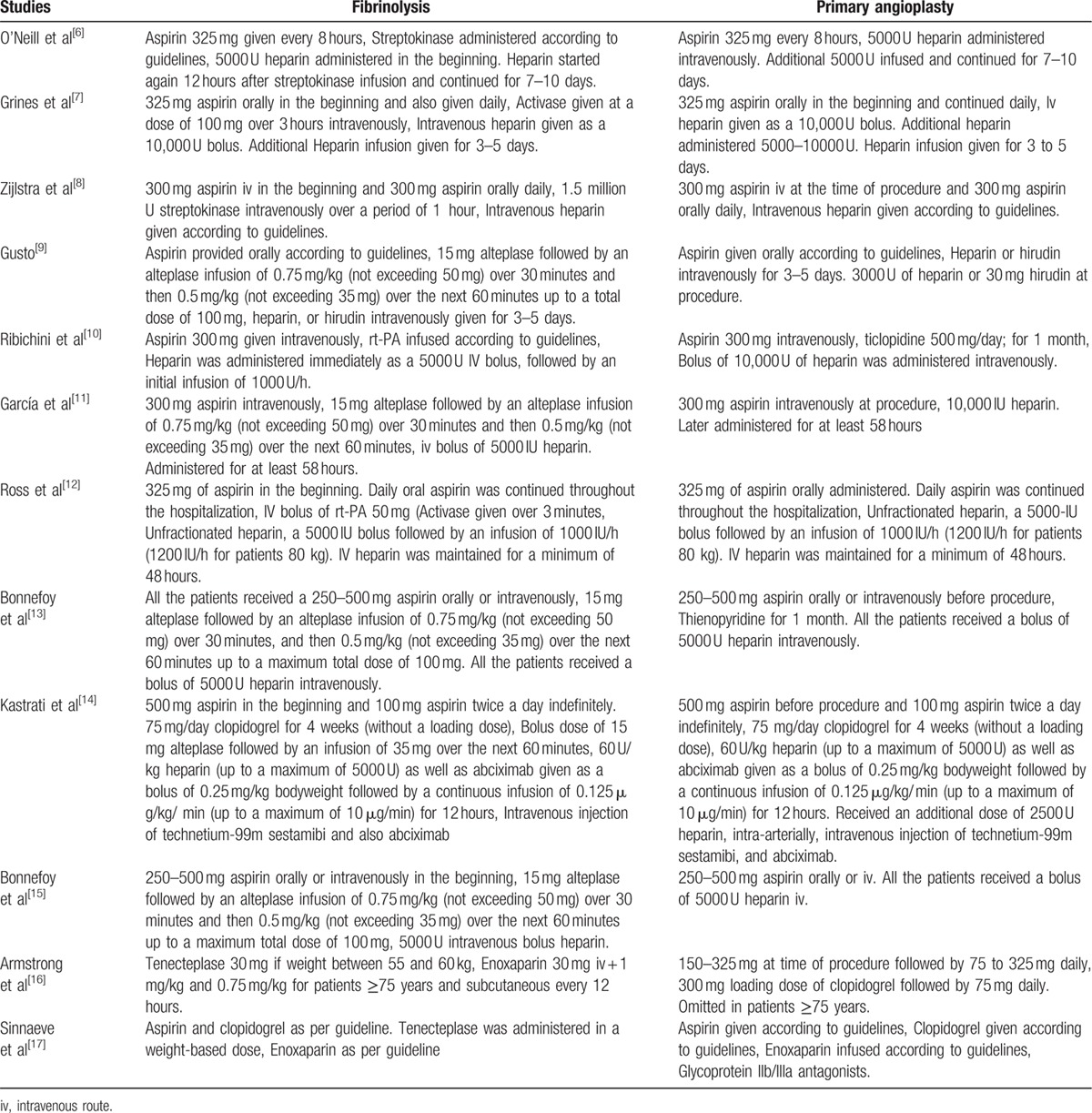
Brief summary of the antiplatelet and antithrombotic medications used by patients randomized to fibrinolysis and primary angioplasty.

### 2.4 Data extraction and quality assessment

Two authors (P.K.B and G.J) independently reviewed and assessed the trials included in this study. Information and data regarding the type of study, the total number of participants randomized to fibrinolysis, the total number of participants randomized to PPCI, the patients’ enrollment period, the antiplatelet and anticoagulants agents used before and during the reperfusion therapies, respectively, the bleeding events and other clinical outcomes reported, and the follow-up periods reported in each eligible trial were systematically extracted. If any of these 2 authors disagreed about including certain data, disagreements were discussed and finally a decision was reached. However, if they could not reach a consensus, a final decision was made by the third author (M.H.C). The 6 main components recommended by the Cochrane Collaboration were taken into consideration while assessing the risk of bias among the trials included in this study^[4]^ whereby a score ranging from 0 to 12 points was allocated to each trial depending on whether each of the 6 components mentioned above corresponded to a low risk, moderate risk, or high risk of bias.

Three studies were allocated a score of 8, 5 studies were allocated a score of 10, and 4 studies were allocated a score of 11. These scores have been listed in Table 2.

**Table 2:**
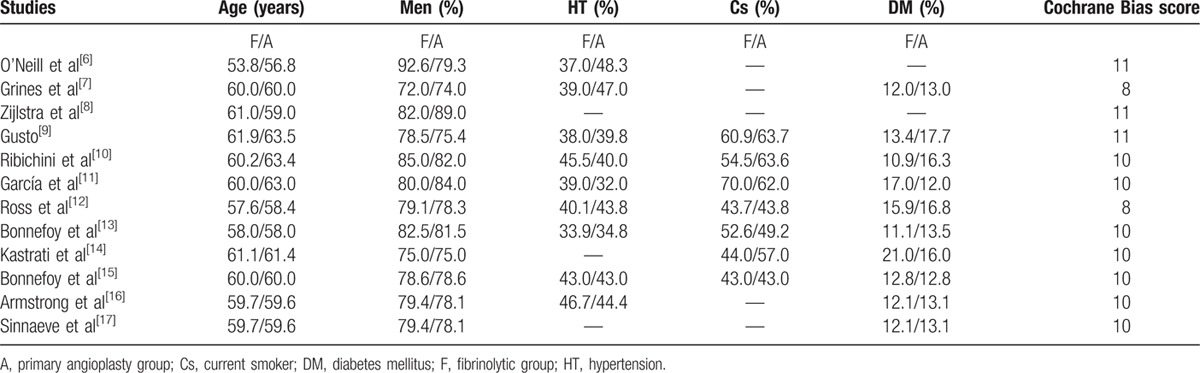
The baseline features of the included studies.

## 3 Outcomes

General bleeding complications included major and minor bleeding, intracranial and nonintracranial hemorrhages, gastrointestinal bleeding, bleeding from other sites, and bleeding that required transfusion of red cells from the period of hospitalization to up to 30 days were considered as the primary clinical outcomes in this study. Intracranial and nonintracranial bleeding were also analyzed separately. Secondary outcomes included death, reinfarction, stroke, and shock. The primary clinical outcomes reported among all the trials have been listed in Table 3.

**Table 3:**
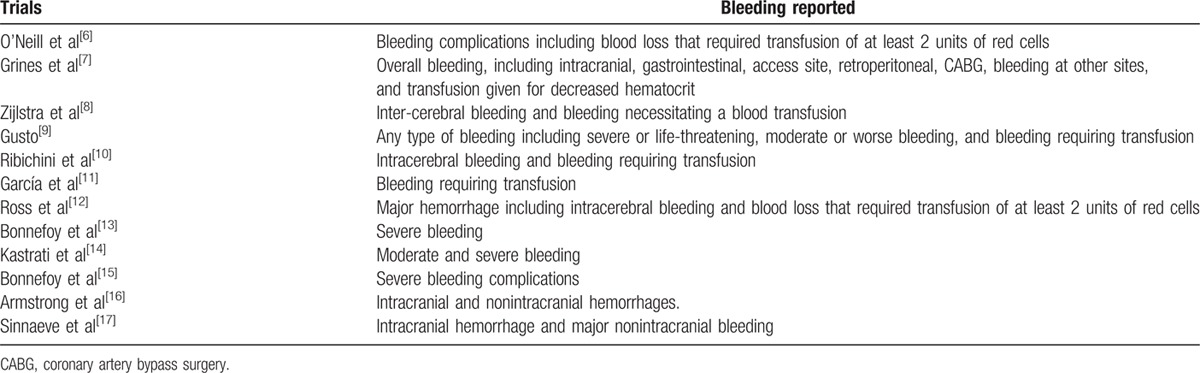
Types of bleeding reported.

Five studies reported in-hospital bleeding, 1 study reported bleeding within 2 weeks, and the remaining 6 studies reported bleeding within or at 30 days.

## 4 Statistical analysis

The PRISMA (Preferred Reporting Items for Systematic Reviews and Meta-Analyses) statement was considered for this systematic review and meta-analysis of RCTs.^[5]^ All authors had full access to and take full responsibility for the integrity of the data. Assessment of heterogeneity during the subgroup analysis was based on the Cochrane Q-statistic whereby a *P* value of ≤0.05 was considered statistically significant, whereas a *P* value of >0.05 was considered statistically insignificant. Moreover, the *I*^2^-statistic test was also considered during the assessment of heterogeneity across the subgroups whereby an *I*^2^ value of 0% indicated no or very low heterogeneity, and an increasing percentage of *I*^2^ indicated increasing heterogeneity. For a better analysis, a fixed effect model was used if *I*^2^ was <50% and a random effect model was used if *I*^2^ was >50%. Funnel plots were used to visually assess publication bias. OR with 95% CIs were calculated for categorical variables and the pooled analyses were performed using the latest version of RevMan 5.3 software. Ethical approval was not required for this type of study.

## 5 Results

### 5.1 Search strategy and analyzed studies

**Figure 1:**
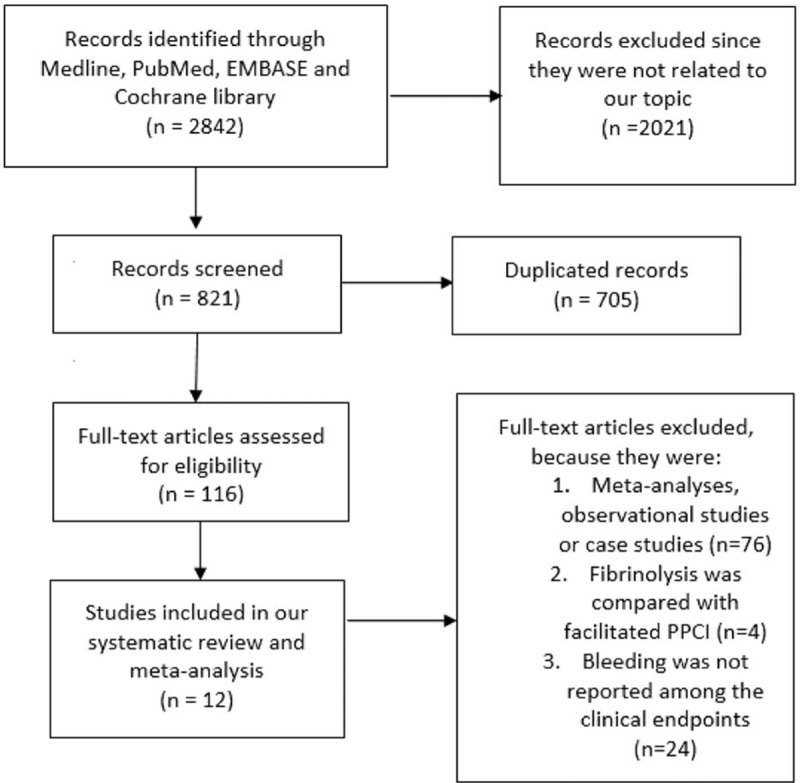
Flow diagram for the study selection.

Two thousand eight hundred forty-two articles were obtained from Medline, PubMed, EMBASE, and the Cochrane databases. Two thousand seven hundred twenty-six articles were eliminated, as they were either not related to our topic or they were duplicates. Among the remaining 116 articles, another 76 articles were eliminated, as they were meta-analyses, case studies, or observational studies. A further 4 articles were eliminated, as they involved facilitated PPCI. When screening studies for inclusion, 36 studies met the pre-defined inclusion criteria of having a randomized comparator group (comparing fibrinolytic therapy with primary angioplasty) between these patients with STEMI. However, because 24 among these 36 studies did not report bleeding complication as one of their clinical outcomes, these studies were strictly excluded. Finally, 12 studies were included in this meta-analysis. The flow diagram for the study selection has been represented in Fig. 1.

The 12 studies included in this meta-analysis reported bleeding complications (primary outcomes) from the period of hospitalization to up to 30 days after reperfusion therapy. Secondary clinical endpoints included all-cause mortality, reinfarction, stroke, and shock. The oldest trial included in this study was published in the year 1986, while the most recent trial included was published in the year 2014. One study was published in the year 1986,^[6]^ 2 in the year 1993,^[7,8]^ 1 in the year 1997,^[9]^ 1 in the year 1998,^[10]^ 2 in the year 1999,^[11,12]^ 2 in the year 2002,^[13,14]^ 1 in the year 2005,^[15]^ 1 in the year 2013,^[16]^ and another one in the year 2014.^[17]^ The general features of the trials included in this study have been represented in Table 4.

**Table 4:**
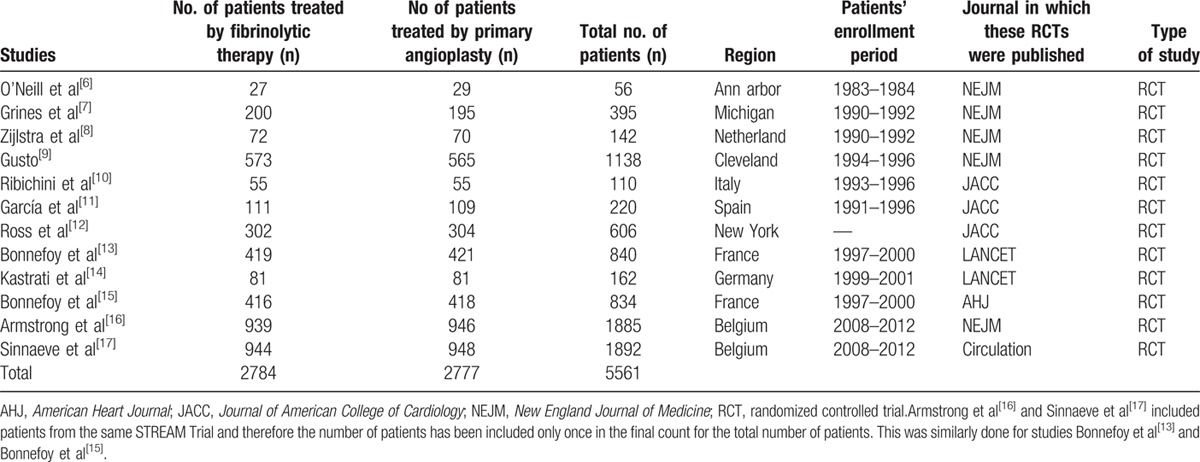
General features of the included studies.

Patient enrollments started from the year 1983 in certain trials until the year 2012 in other trials. Randomization of the patients to either fibrinolytic therapy or primary angioplasty was performed in several medical centers mainly from European countries such as Spain, France, Germany, Belgium, and Netherland.

A total number of 5561 patients (2784 patients from the fibrinolysis group and 2777 patients from the PPCI group) were included in this meta-analysis. All patients provided signed consents. The general features of these trials have been listed in Table 4.

### 5.2 Baseline characteristics

Table 2 reports the demographic features, including mean age, percentage of males, percentage of patients suffering from hypertension, diabetes, and those patients who are heavy smokers. Patients had an average age of 60 years in both the fibrinolytic and angioplasty groups. Except from 1 study, the percentage of males in the 2 reperfusion groups were almost similar. The percentages of patients suffering from hypertension, diabetes mellitus, and those patients who had a history of smoking were almost indifferent between these 2 groups. Overall, there was no significant difference in the baseline features between the fibrinolytic and angioplasty groups. These characteristics have been summarized in Table 2.

### 5.3 Bleeding risk: analysis of the 5561 patients

**Figure 2:**
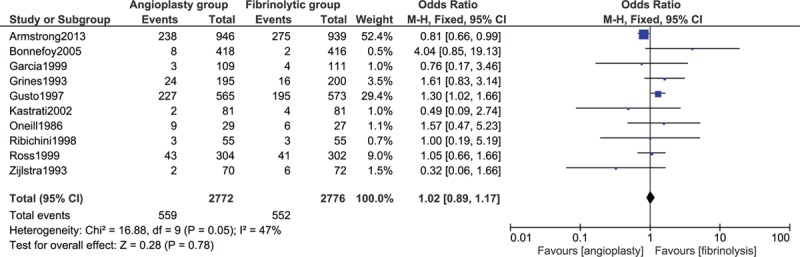
General bleeding complications between fibrinolysis and primary percutaneous coronary intervention.

The pooling analysis showed no significant difference in the general bleeding complications between patients who underwent reperfusion with fibrinolytic therapy and primary angioplasty with OR 1.02; 95% CI 0.89 to 1.17, *P* = 0.78. This result has been represented in Fig. 2.

**Figure 3:**
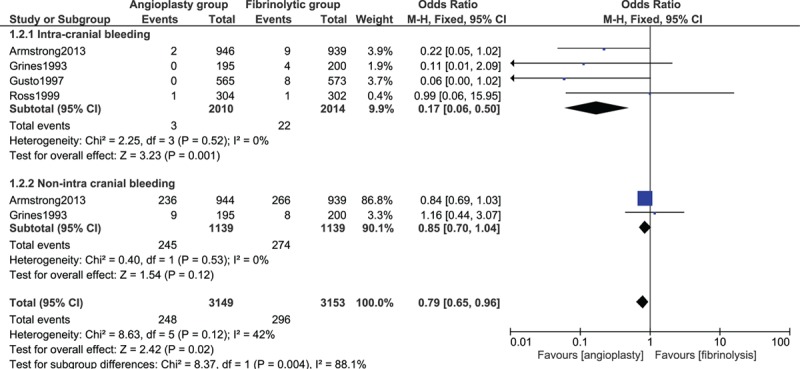
Intracranial and nonintra cranial bleeding between fibrinolytic therapy and primary percutaneous coronary intervention.

When intracranial and extracranial bleeding were analyzed separately, PPCI was associated with a significantly lower rate of intracranial bleeding than fibrinolysis with OR 0.17; 95% CI 0.06 to 0.50, *P* = 0.001. Nonintracranial bleeding also favored PPCI with OR 0.85; 95% CI 0.70 to 1.04, *P* = 0.12; however, this result was not statistically significant. Results assessing intracranial and nonintracranial bleeding have been illustrated in Fig. 3.

### 5.4 Secondary outcomes

**Figure 4:**
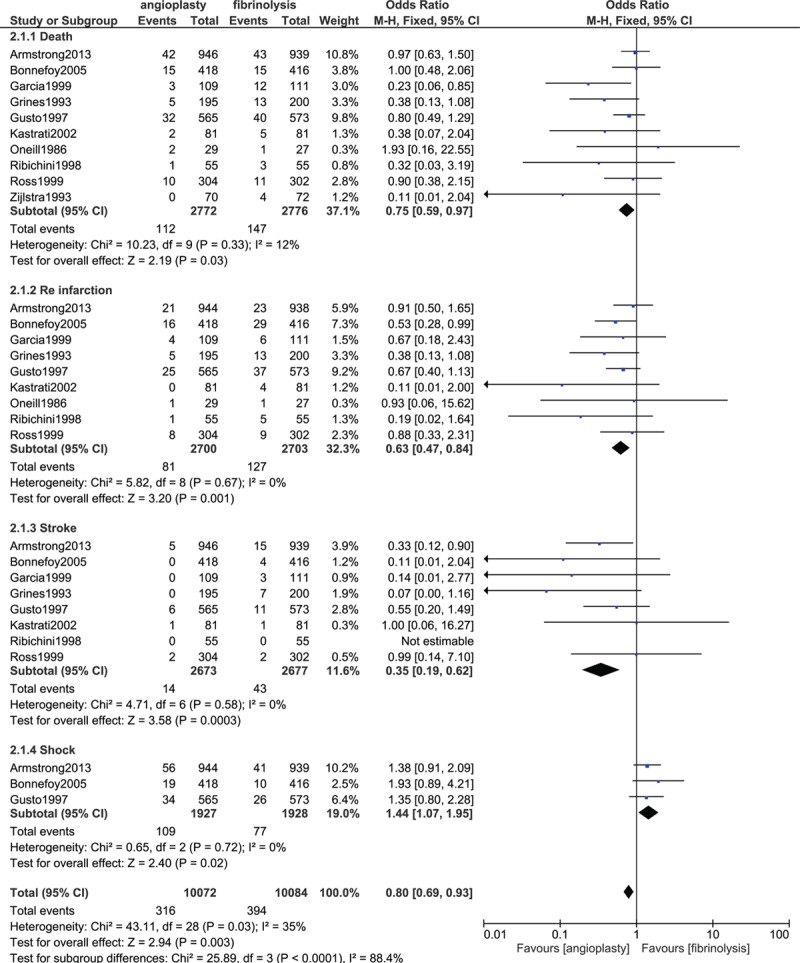
Secondary endpoints analyzed between fibrinolysis and primary percutaneous coronary intervention.

According to the patients analyzed, mortality was significantly higher in the fibrinolysis group with OR 0.75; 95% CI 0.59 to 0.97, *P* = 0.03. Reinfarction also significantly favored PPCI with OR 0.63; 95% CI 0.47 to 0.84, *P* = 0.001. Stroke was significantly lower in the PPCI group with OR 0.35; 95% CI 0.19 to 0.62, *P* = 0.0003. However, our results showed that shock significantly favored fibrinolysis with OR 1.44; 95% CI 1.07 to 1.95, *P* = 0.02. Results reporting the secondary outcomes have been illustrated in Fig. 4.

**Figure 5:**
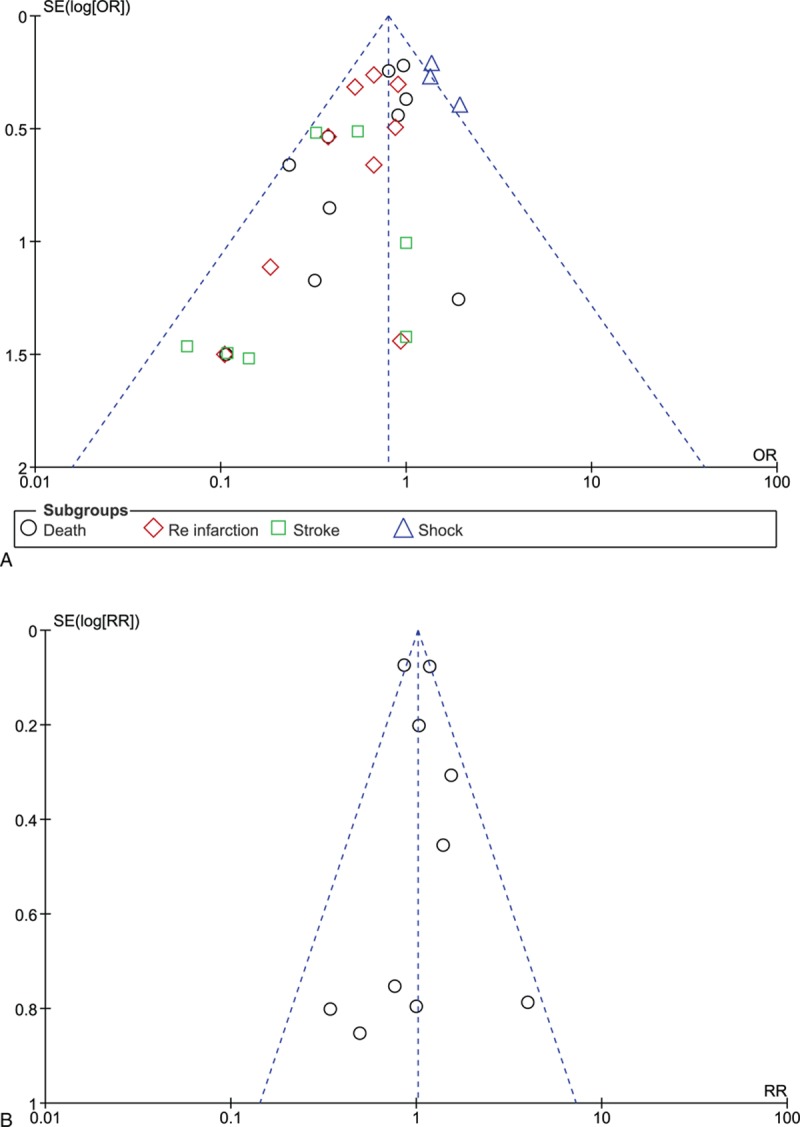
(A) and (B) Funnel plots representing sensitivity analysis.

For all of the above analyses, sensitivity analyses yielded consistent results. On the basis of a visual inspection of the funnel plots that assessed primary and secondary outcomes in this study, there has been no evidence of publication bias among the included studies. The funnel plots have been illustrated in Fig. 5A and B.

## 6 Discussion

Patients with STEMI often undergo reperfusion therapy by fibrinolysis or PPCI. However, both reperfusion therapies are associated with bleeding complications. Hence, we have conducted this meta-analysis including 5561 randomized patients in order to compare the bleeding events between these 2 reperfusion therapies so as to know which one is safer during a 30-day period after reperfusion.

Despite the differences in antiplatelet and anticoagulation regimens between these 2 groups, fibrinolytic therapy and primary angioplasty were associated with almost similar overall bleeding complications (including all types of bleeding combined together). However, when bleeding events were further classified into intracranial and nonintracranial bleeding, fibrinolysis was associated with a significantly higher rate of intracranial bleeding than PPCI. Other secondary outcomes including death, reinfarction, and stroke significantly favored PPCI. However, shock significantly favored fibrinolysis.

Very few research reported bleeding events between these 2 reperfusion therapies. Most of the studies mainly focused on the impact of fibrinolytic therapy and primary angioplasty on mortality in patients with STEMI. The randomized trial of direct coronary angioplasty versus streptokinase in patients with acute MI conducted by Ribeiro et al^[18]^ did not report any bleeding complication between these 2 reperfusion therapies and stated that not even a single patient in either group required blood transfusion during this in-hospital follow-up period. However, another study by Mehta et al^[19]^ comparing fibrinolytic therapy with primary angioplasty showed a decreased in mortality and reinfarction in the angioplasty group with no change in other outcome measures, in elderly patients with STEMI.

This current study involved only published trials. However, results from an observational study showed a major bleeding (3.2%) associated with thrombolytic therapy and (4.2%) associated with primary angioplasty in low-volume primary angioplasty hospitals.^[20]^ But, in intermediate and high volume hospitals, the major bleeding rates were 4.3% and 3.8%, and 4.0% and 3.6%, in the thrombolytic and angioplasty groups, respectively.

In addition, the comparison of tenecteplase with rt-PA also showed that tenecteplase did not increase intracranial hemorrhage but was associated with less noncerebral bleeding especially in high-risk patients.^[2]^ However, as our study compared fibrinolytic therapy with primary angioplasty, our results were completely different. A meta-analysis conducted by Kwok et al^[3]^ using data from 42 studies concluded that major bleeding after PCI was independently associated with a 3-fold increase in mortality and major adverse cardiac outcomes, which was also different from the results of this current study. But, however, their study was not comparing PCI with fibrinolytic therapy.

These 2 types of reperfusion therapies in patients with STEMI have their own particular advantages. Fibrinolytic therapy is a reperfusion therapy that is easier and does not require any type of surgery. This kind of reperfusion therapy is more common in PCI-noncapable centers.

On the contrary, PPCI is an invasive procedure that is not as easy as the fibrinolytic therapy, and requires skills and can only be performed by an experienced interventionist.

Because the resistance of cross-linked fibrin to fibrinolysis is time-dependent, fibrinolysis is most effective when given within the first four hours after the onset of symptoms, particularly within the first 70 minutes.^[21,22]^ Factors such as delayed to be transported to the hospital, an unclear or unknown patient history or, contraindication to fibrinolysis, could limit the use of this reperfusion therapy in many medical centers. Thus, guidelines recommend primary angioplasty as a better option even if both treatment regimens are almost similar.^[1]^

## 7 Limitations

Due to a smaller population size, the result of this study might be restricted to some extent. Moreover, 1 study did not report all-cause mortality, and therefore, data for cardiac mortality were used instead. This could have an effect on our results. Different follow-up periods ranging from in-hospital follow-up to a follow-up period of 30 days were reported. Combining and comparing studies with different follow-up periods altogether could also be a limitation in this study.

## 8 Conclusion

According to the results of this study, even if the rate of nonintracranial bleeding was not statistically significant between these 2 reperfusion therapies, fibrinolytic therapy was associated with a significantly higher rate of intracranial bleeding than PPCI. In addition, PPCI was associated with a significantly lower rate of death, reinfarction, and stroke. Therefore, PPCI should be recommended in patients with STEMI, especially in PCI-capable hospitals. Fibrinolysis should only be reserved for non-PCI capable hospitals.
